# Users’ Perceived Service Quality of National Telemedicine Services During the COVID-19 Pandemic in Bangladesh: Cross-Sectional Study

**DOI:** 10.2196/46566

**Published:** 2024-12-23

**Authors:** Fatema Khatun, Novel Chandra Das, Md Rakibul Hoque, Kazi Nazmus Saqeeb, Monjur Rahman, Kyung Ryul Park, Sabrina Rasheed, Daniel D Reidpath

**Affiliations:** 1 Health Systems and Population Studies Division, International Centre for Diarrheal Disease Research, Bangladesh (icddr,b), 68, Shaheed Tajuddin Ahmed Sharani, Mohakhali, Dhaka, 1212, Bangladesh, 880 1715287117; 2 Department of Management Information Systems, University of Dhaka, Dhaka, Bangladesh; 3 Nutrition Research Division, International Centre for Diarrheal Disease Research, Bangladesh (icddr,b), Dhaka, Bangladesh, Bangladesh; 4 Maternal and Child Health Division, International Centre for Diarrheal Disease Research, Bangladesh (icddr,b), Dhaka, Bangladesh, Bangladesh; 5 Graduate School of Science and Technology Policy, Korea Advanced Institute of Science and Technology, Daejeon, Republic of Korea; 6 The Institute for Global Health and Development, Queen Margaret University, Edinburgh, United Kingdom

**Keywords:** telemedicine, COVID-19, LMIC, low- and middle-income countries, Bangladesh, service quality, user satisfaction, structural equation modeling, digital health

## Abstract

**Background:**

COVID-19 created an opportunity for using teleconsultation as an alternative way of accessing expert medical advice. Bangladesh has seen a 20-fold increase in the use of teleconsultation during the pandemic.

**Objective:**

The aim of our study was to assess the influence of service quality and user satisfaction on the intention to use teleconsultation in the future among users of national teleconsultation services during the pandemic.

**Methods:**

A cross-sectional survey was conducted in 2020 among users of the national teleconsultation service—Shastho Batayon for acute respiratory infection. A validated mobile health service quality model based on structural equation modeling and confirmatory factor analysis was used to analyze the data with SmartPLS (version 3.0).

**Results:**

Among the 2097 study participants, 1646 (78.5%) were male, 1416 (67.5%) were aged 18‐39 years, 1588 (75.7%) were urban residents, 1348 (64.2%) had more than 10 years of schooling, and 1657 (79%) were from middle-income households. From a consumer perspective, the quality of the service platform (β=.946), service interaction (β=.974), and outcome (β=.955) contributed to service quality. Service quality was positively associated with user satisfaction (β=.327; *P*<.001) and intention to use teleconsultation services (β=.102; *P*<.001). User satisfaction was positively associated with the intention to use teleconsultation services (β=.311; *P*<.001).

**Conclusions:**

The increase in the use of teleconsultation during the pandemic indicated that such services were potentially used for emergencies. However, the future use of teleconsultation will be dependent on the quality of service and user satisfaction. Our findings are relevant for low-income contexts where teleconsultation services are used to address gaps in service delivery.

## Introduction

### Background

Globally, 58% of countries have adopted telemedicine to address the disruption of health care services during the COVID-19 pandemic [1]. Telemedicine facilitates the delivery of remote health care services at low cost [2] and has shown to enable quick and equitable access to health care for patients in hard-to-reach areas [3]. Telemedicine has been effectively used to provide health services during previous SARS-CoV-2 and Middle East respiratory syndrome outbreaks [4], and during the COVID-19 pandemic, the use of telemedicine has increased globally to minimize the risks of viral transmission [5,6]. Researchers around the world reported widespread use of telemedicine in many low- and middle-income countries (LMICs) where the provision of protective gear, vaccines, and hospital facilities was inadequate during the pandemic [7-13].

In Bangladesh, there was severe disruption of primary health care provision during the early stage of the pandemic, as health systems responded to lockdown measures, deaths among health care providers, and the need to mitigate and limit the spread of COVID-19 [14]. One of the response measures by the government of Bangladesh was to make the existing national teleconsultation platform “Shastho Batayon 16263” toll-free [15]. As travel restriction was in place and access to face-to-face consultation was limited, calls to the national teleconsultation platform increased 20-fold during the pandemic [16]. However, there were concerns about the negative impact of the sudden increase in demand for teleconsultation services on the existing infrastructure and human resources of Shastho Batayon (SB) [17] and whether any compromise in terms of service quality of the platform would negatively impact patient satisfaction and intention to avail teleconsultation services in the future [18].

Although the promises are offered by telemedicine in low-resource settings, ensuring the quality of telemedicine services is a major challenge [19]. Despite the challenges of quality, however, there are benefits of using telemedicine during a pandemic such as COVID-19 [20], and it is likely that many patients and providers will continue to use this service in the postpandemic period [21]. Hence, it is important to understand how the service quality of telemedicine affects patient satisfaction and intention to use the service in the future [22].

The World Health Organization’s guiding principles for implementing telemedicine services during COVID-19 listed “user satisfaction” as one of the key components for evaluation [23]. In health care, service quality is a major indicator of patient satisfaction [24-27]. Satisfaction is associated with increased user retention and intention to use the service in the future [24,25,28].

There are a number of theories and models that address quality dimensions of information and communication technology–based services. A systematic review of the service quality models identified 30 models, which described that the most common dimensions were tangibles, reliability, responsiveness, empathy, and assurance [29]. In a developing country context, the mobile health (mHealth) service quality model tested by Akter et al [30] measures the association between service quality and satisfaction on the intention to use the service in the future. This conceptual model describes platform quality, interaction quality, and outcome quality as primary dimensions of service quality for mHealth and proposes a direct association between service quality and patient satisfaction and an indirect association between service quality and intention to continue to use the service through patient satisfaction [30]. The conceptual model has been previously validated for Bangladesh. Using the model for mHealth service quality, we aimed to assess the influence of service quality and satisfaction on the intention to use SB services in the future among the users who availed the service during the pandemic. The insights from this study will significantly contribute to the sustainability of national teleconsultation services during future health emergency situations and beyond.

### Theoretical Framework

For this study, we used the mHealth service quality model [30] to design the study. A number of empirical studies have used the mHealth service quality model to describe the association between service quality and users’ intention to continue using eHealth services [24,30-32], but the model by Akter et al [30] is chosen, as it was used to teleconsultation in the context of LMICs. The conceptual framework and the hypotheses are detailed in the following subsections.

### Conceptual Model: A Hierarchical, Multidimensional Service Quality Model

**Figure 1.**
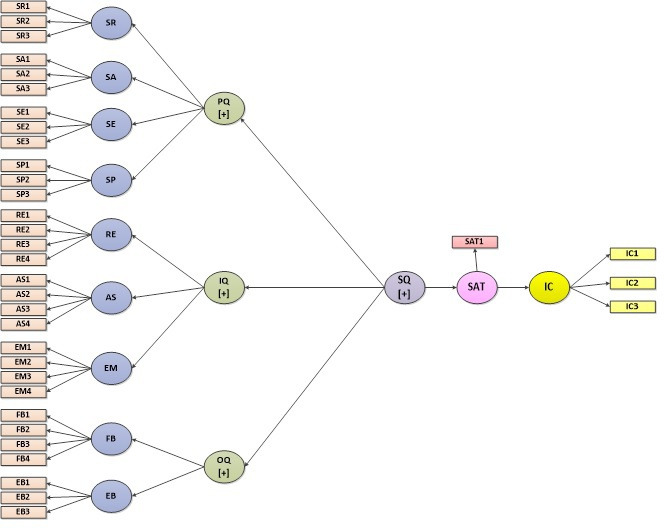
Conceptual framework of service quality conceptual model for national teleconsultation services users during the COVID-19 pandemic in Bangladesh, October-December 2020 (N=2097). AS: assurance; EB: emotional benefit; EM: empathy; FB: functional benefit; IC: intension; IQ: interaction quality; OQ: outcome quality; PQ: platform quality; RE: responsiveness; SA: systems availability; SAT: satisfaction; SE: systems efficiency; SP: systems privacy; SQ: service quality; SR: systems reliability.

The conceptual model (Figure 1) of service quality used in this study postulates a direct relationship between service quality and satisfaction and that service quality indirectly works on intention to use, mediated by satisfaction [33-36]. In the service quality model, service quality comprises 3 primary dimensions (platform quality, interaction quality, and outcome quality) and 9 subdimensions. The dimension platform quality has systems reliability, systems availability, systems efficiency, and systems privacy as subdimensions; the interaction quality dimension has responsiveness, assurance, and empathy as subdimensions; and the outcome quality dimension has functional benefit and emotional benefit as subdimensions.

### Definition of the Constructs

The definitions of constructs such as platform quality, interaction quality, outcome quality, user satisfaction, and intention to continue are detailed in Multimedia Appendix 1.

### Hypotheses

In this study, we tested 3 hypotheses:

- Hypothesis 1: SB service quality has a positive impact on user satisfaction.
- Hypothesis 2: User satisfaction with SB is positively associated with the intention to use the services in the future.
- Hypothesis 3: SB service quality is positively associated with the intention to use the service in the future.

## Methods

### Study Settings and Participants: SB

The Ministry of Family and Welfare of Bangladesh, in an effort to facilitate access to health services, runs a national health helpline with SB through its Management Information System division. The service is available 24/7, and callers can consult doctors and get limited prescriptions and referrals. During the COVID-19 pandemic, the SB number was made toll-free.

We conducted this study among the users of SB from October 19, 2020, to December 31, 2020. Our study participants were those who called SB with their flu-like syndromes and were provided a provisional diagnosis of suspected or confirmed COVID-19. We approached the participants from the random list collected from the Management Information System database who sought teleconsultation services from any geographical area of Bangladesh and were interested to join the study, and those participants were enrolled as study participants. We were provided with a total list of 7400 people, with batches of 100 participants sent out each day from SB, and finally, 2097 participants were enrolled in the study. All the interviews were conducted over the phone.

### Sample Size

To use structural equation modeling (SEM), the number of constructs (latent variables), number of observed variables, and effect size of the loading coefficient with a 95% confidence level and 80% power were considered with a view to calculating the required sample size through Enrico multivariate software [37]. To identify the effect size of the loading coefficient of 0.15, 35 observed variables, and 15 latent variables with 95% confidence level and 80% power, the minimum required sample size was 378. Thus, the study fulfills the requirement of the necessary sample size to apply SEM by collecting information from 2097 participants.

### Data Collection

Within 2 weeks of seeking care from SB by the selected caller, we administered this satisfaction survey about their call to SB. We collected a list of 100 callers (randomly selected) every day based on our selection criteria (suspected or confirmed COVID-19 cases) from October 19, 2020, to December 31, 2020.

The list was then divided and distributed among the data collectors for conducting a telephone survey. Data were collected using a questionnaire previously validated in Bangladesh [32]. All the data were collected through a web-based Android-based data collection app or tool by trained data collectors. In total, 5% of the interviews were randomly rechecked by the supervisor to ensure the data quality and validity of the interviews.

### Ethical Considerations

The study was approved by the institutional review board of the International Centre for Diarrheal Disease Research, Bangladesh (protocol: PR#19091). Informed verbal consent was taken from all the study participants over the phone. In total, 31 study participants’ age was below 18 years so we took informed assent from their guardians over the phone to participate in the study. The privacy, anonymity, and confidentiality of the research data and information were strictly maintained.

### Variables

The questionnaire included questions on user demographics (age, sex, socioeconomic status, and place of residence). To understand users’ perception of service quality, the questionnaire included questions related to systems reliability, availability, efficiency, privacy, responsiveness, assurance, empathy, functional, emotional benefit, and satisfaction along with the intention to continue using the service in the future. A 5-point Likert scale with a range from 1=strongly disagree to 5=strongly agree was used to measure the responses. The statements used in the questionnaire were previously validated in Bangladesh [32]. The questionnaire is available in Multimedia Appendix 2.

### Normality Check

The assessment of data normality was conducted through the estimation of skewness and kurtosis for each measurement item. The skewness values ranged from −0.319 to 0.069, while kurtosis values ranged from −0.543 to 0.671, where values within the range of ±2 for skewness and ±5 for kurtosis are deemed acceptable indicators of normal distribution [38], our dataset exhibited no substantive issues with skewness or kurtosis.

### Data Analysis

From the list of 7400 individual users of SB, we were able to reach only 2163 due to incorrect numbers, switched off phones, unavailable networks, use of neighbors’ or relatives’ phone numbers for SB services, and because callers did not receive our calls. The initial response rate was 29.2%. Of those we were able to reach, 3.9% (n=66) of respondents refused to participate in the study so our final sample was 2097.

SmartPLS (version 3.0) was used to analyze the data. Component-based SEM or partial least squares (PLS) path modeling was used to understand the direct effect between service quality and satisfaction and satisfaction and intention and the indirect effect between service quality and intention. Confirmatory factor analysis (CFA) was used to test the hypotheses. First-order CFA was used to test the validity and reliability of measurement items of the constructs. Second- and third-order CFA were used in the measurement model because there are latent variables that were constructed as dimensions of another variable. To assess the second-order reflective model of service quality, this study used PLS Graph (version 3.0; SmartPLS GmbH) [39] and PLS path modeling with a path weighing scheme for the inside approximation [40-42].

## Results

### Sociodemographic Characteristics of Study Participants

Among the 2097 study participants, the majority were from urban areas (n=1588, 75.7%), had more than 10 years of schooling (n=1348, 64.3%), and were from middle-income households (n=1657, 79%; Table 1). Most of the service recipients were male (n=1646, 78.5%), 67.5% (n=1416) were between 18 and 39 years of age, 63.2% (n=1325) were married, and 37.3% (n=782) had more than 4 members in their household (Table 1).

**Table 1.** Sociodemographic characteristics of the study participants of Shastho Batayon teleconsultation services users during the COVID-19 pandemic in Bangladesh, October-December 2020 (N=2097).

Characteristics		Values, n (%)
**Age (years)**		
	<18	31 (1.5)
	18‐39	1416 (67.5)
	40‐59	579 (27.6)
	≥60	71 (3.4)
**Sex**		
	Male	1646 (78.5)
	Female	451 (21.5)
**Residence**		
	Village	290 (13.8)
	Semiurban	219 (10.4)
	Urban	1588 (75.7)
**Education (years)**		
	≤5	331 (15.8)
	6‐10	418 (19.9)
	>10	1348 (64.3)
**Marital status**		
	Unmarried	732 (34.5)
	Married	1325 (63.2)
	Widowed	37 (1.7)
	Divorced	3 (0.1)
**Occupation**		
	Business	263 (12.5)
	Unskilled labor	18 (0.86)
	Skilled labor	30 (1.4)
	Service holder	1035 (49.3)
	Agriculture worker	33 (1.6)
	Student	252 (12)
	Housewife	255 (12.2)
	Unemployed	118 (5.6)
	Others	93 (4.4)
**Family member**		
	≤4	1315 (62.7)
	>4	782 (37.3)
**Monthly household expenditure (US $)**		
	>500	182 (8.7)
	50‐500	1657 (79)
	<50	258 (12.3)

### Assessment of the Measurement Model

The study assesses the psychometric properties of the first-order measurement model by examining reliability, convergent validity (Table 2), and discriminant validity (Table 3). Cronbach α, composite reliabilities, and average variance extracted from the data exceeded the cutoff values of 0.5, 0.7, and 0.5, respectively (Table 2), which indicate adequate scale reliability and validity [40,43]. The model was considered satisfactory in terms of reliability, convergent validity, and discriminant validity. The result showed that the composite reliabilities and average variance extracted from the second- and third-order scales were greater than 0.8 and 0.5, respectively, which indicated that the higher-order measures were reliable (Table 2).

**Table 2.** Confirmatory factor analysis and psychometric properties of the hierarchical service quality scale^a^.

Factor and items		Loadings	Cronbach α	First-order constructs		Second-order constructs			Third-order constructs		
				CR^b^	AVE^c^	Constructs	CR	AVE	Constructs	CR	AVE
**Systems reliability (SR)**			0.950	0.969	0.911	Platform quality	0.956	0.647	Service quality	0.985	0.685
	SR1	0.942									
	SR2	0.971									
	SR3	0.951									
**Systems availability (SA)**			0.872	0.922	0.797	—^d^	—	—	—	—	—
	SA1	0.908									
	SA2	0.927									
	SA3	0.841									
**Systems efficiency (SE)**			0.90	0.938	0.834	—	—	—	—	—	—
	SE1	0.901									
	SE2	0.932									
	SE3	0.906									
**Systems privacy (SP)**			0.923	0.951	0.867	—	—	—	—	—	—
	SP1	0.946									
	SP2	0.943									
	SP3	0.903									
**Responsiveness (RE)**			0.920	0.943	0.806	Interaction quality	0.975	0.768	—	—	—
	RE1	0.901									
	RE2	0.909									
	RE3	0.892									
	RE4	0.890									
**Assurance (AS)**			0.936	0.954	0.839	—	—	—	—	—	—
	AS1	0.903									
	AS2	0.916									
	AS3	0.926									
	AS4	0.918									
**Empathy (EM)**			0.952	0.966	0.875	—	—	—	—	—	—
	EM1	0.946									
	EM2	0.957									
	EM3	0.940									
	EM4	0.899									
**Functional benefit (FB)**			0.953	0.966	0.876	Outcome quality	0.979	0.869	—	—	—
	FB1	0.926									
	FB2	0.933									
	FB3	0.942									
	FB4	0.943									
**Emotional benefit (EB)**			0.961	0.975	0.928	—	—	—	—	—	—
	EB1	0.958									
	EB2	0.965									
	EB3	0.966									
Satisfaction	—	1	—	1	1	—	—	—	—	—	—
**Intention to continue (IC)**			—	0.951	0.866	—	—	—	—	—	—
	IC1	0.935									
	IC2	0.934									
	IC3	0.922									

a Convergent validity: loadings >0.70. Scale reliability: CR>0.80, AVE>0.50.

b CR: composite reliability.

c AVE: average variance extracted.

d Not applicable.

**Table 3.** Correlation of first-order constructs^a^.

	AS^b^	EB^c^	EM^d^	FB^e^	IC^f^	RE^g^	SA^h^	SAT^i^	SE^j^	SP^k^	SR^l^
AS	0.916	—^m^	—	—	—	—	—	—	—	—	—
EB	0.852	0.963	—	—	—	—	—	—	—	—	—
EM	0.899	0.858	0.936	—	—	—	—	—	—	—	—
FB	0.895	0.935	0.907	0.936	—	—	—	—	—	—	—
IC	0.761	0.824	0.753	0.795	0.930	—	—	—	—	—	—
RE	0.860	0.793	0.856	0.834	0.709	0.898	—	—	—	—	—
SA	0.637	0.635	0.628	0.657	0.562	0.697	0.893	—	—	—	—
SAT	0.324	0.308	0.323	0.333	0.311	0.269	0.173	1.000	—	—	—
SE	0.817	0.786	0.795	0.827	0.712	0.786	0.669	0.304	0.913	—	—
SP	0.682	0.621	0.670	0.682	0.567	0.662	0.568	0.230	0.755	0.931	—
SR	0.773	0.783	0.766	0.798	0.689	0.751	0.659	0.301	0.801	0.603	0.955

a Discriminant validity: square root of average variance extracted on the diagonal>correlation coefficients.

b AS: assurance.

c EB: emotional benefit.

d EM: empathy.

e FB: functional benefit.

f IC: intension.

g RE: responsiveness.

h SA: systems availability.

i SAT: satisfaction.

j SE: systems efficiency.

k SP: systems privacy.

l SR: systems reliability.

m Not applicable.

### Structural Equation Modeling

**Figure 2.**
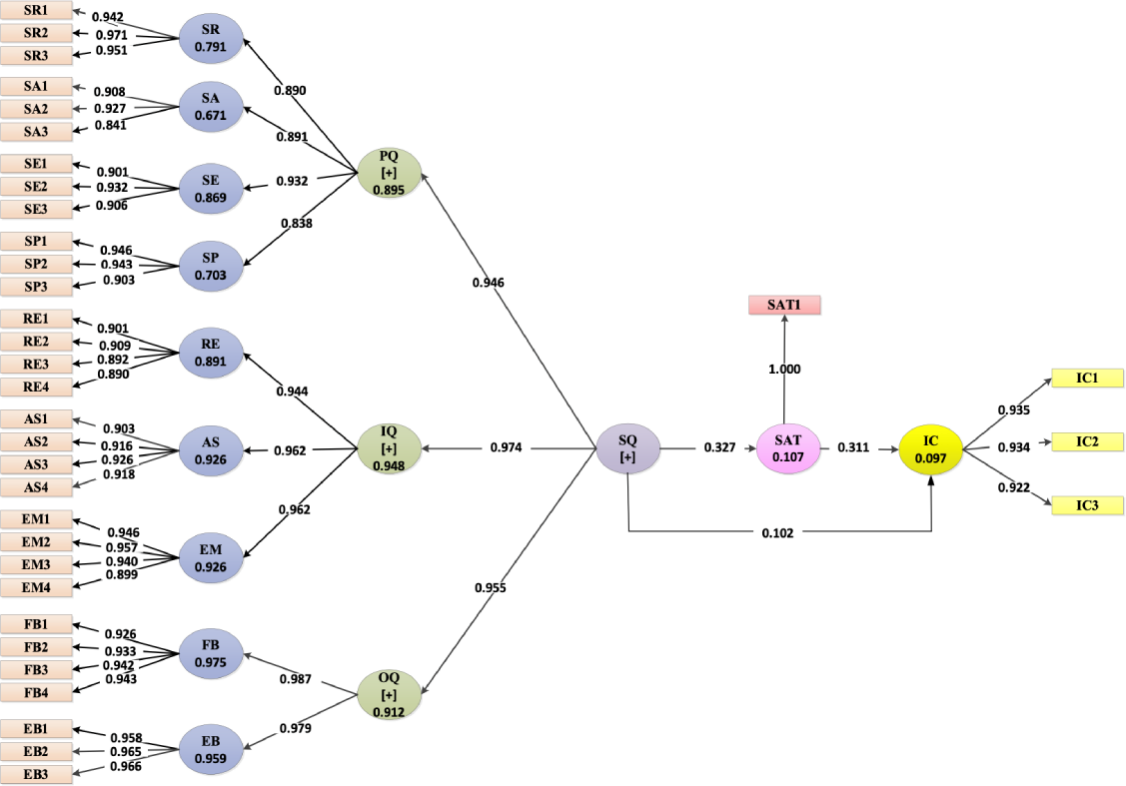
Path coefficients of the research model for national teleconsultation services users during the COVID-19 pandemic in Bangladesh, October-December 2020 (N=2097). AS: assurance; EB: emotional benefit; EM: empathy; FB: functional benefit; IC: intension; IQ: interaction quality; OQ: outcome quality; PQ: platform quality; RE: responsiveness; SA: systems availability; SAT: satisfaction; SE: systems efficiency; SP: systems privacy; SQ: service quality; SR: systems reliability.

**Figure 3.**
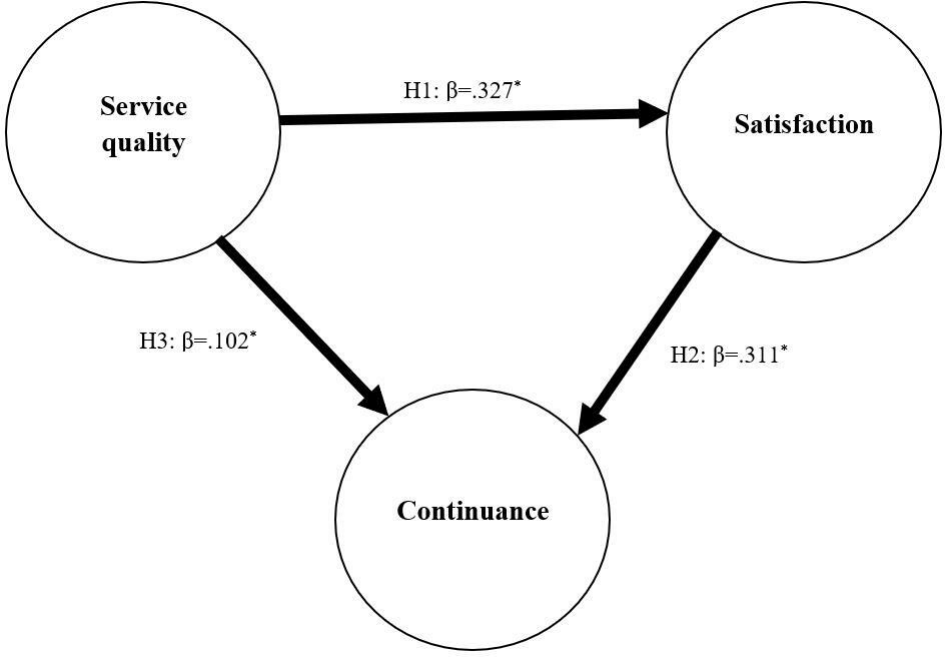
Testing hypotheses regarding the association between Shastho Batayon service quality, user satisfaction, and continuance intention, October-December 2020 (N=2097). H: hypothesis. *P<.001.

Our results showed that the third-order construct, service quality, was associated with the second-order constructs—platform quality (β=.946), interaction quality (β=.974), and outcome quality (β=.955), which explained 90%, 95%, and 91% of the overall quality variance, respectively (Figure 2). The second-order construct, outcome quality, was significantly associated with their first-order constructs—functional benefit (β=.987) and emotional benefit (β=.979; Figure 2). All the path coefficients from service quality to second-order and third-order components were significant at *P*<.001 (Figure 3). Therefore, we found that the 31 items, grouped into 9 factors, can be used to measure the overall service quality of SB teleconsultation services.

### Hypotheses Testing

- Hypothesis 1: The β from service quality to satisfaction was .327, indicating that SB service quality was positively associated with user satisfaction.
- Hypothesis 2: The β from satisfaction to intention to continue use was .311, indicating that user satisfaction with SB was positively associated with the intention to use the services in the future.
- Hypothesis 3: The β from service quality to continuance intention was .102, indicating that SB service quality was associated with the intention to use the service in the future.

## Discussion

### Principal Findings

Our study describes the use of toll-free national teleconsultation services (SB) during the COVID-19 pandemic using a service quality model validated in Bangladesh [16]. In our analysis, we assessed the impact of service quality on user satisfaction and intention to use SB services in the future. During the pandemic, with the rapid increase in the number of calls to SB, the service provision had to be scaled up rapidly to cater to the needs of the population. However, our study indicates that teleconsultation services such as SB have to pay attention to service quality if they want the service recipients to continue to avail the services beyond the pandemic. In the context of Bangladesh, researchers have described [44,45] factors such as usefulness, perceived reliability, price value, technology anxiety, expectations, performance, disconfirmation, and enjoyment influencing the adoption, satisfaction, and continuance of mHealth and telemedicine services in Bangladesh [44,46-49], although in most cases, the studies did not use validated scales for service quality. In most cases, the mHealth interventions studied were small-scale or were being piloted among both rural and urban populations. Only 1 study was conducted to conceptualize and validate service quality scales [31]. To our knowledge, this is the first study to assess the association between service quality and both user satisfaction and intention to use the service in the future for national teleconsultation service.

The service quality of SB was positively associated with user satisfaction. This finding emphasizes the importance of looking at the SB service from the viewpoint of “systems as service” as described by earlier researchers [30]. In terms of the 3 subdomains of service quality, such as platform quality, interaction quality, and outcome quality, SB services were perceived to provide the needed health care during the COVID-19 pandemic. Although in other studies, the price of the service was an important determinant of user satisfaction along with service quality [50], calls to SB were made toll-free during the pandemic period, which could have contributed to both increase in the number of calls [51] and user satisfaction. It is important that the insights related to the rapid scaling up of the SB services while maintaining service quality during emergencies are systematically recorded and included in the standard operating procedures in the future.

We found that service quality and user satisfaction were important determinants of the intention to use SB services in the future. Similar findings about the association between telemedicine service quality and user satisfaction on intention to use the services have been reported from both developed and developing countries [52,53]. Whether users will continue to use SB services for their future health care needs after the COVID-19 pandemic will depend on both service quality and user satisfaction. A sound technological platform is a necessity for providing reliable teleconsultation services. However, service quality also depends on the quality of interaction between the service providers and the users. Interaction quality could be improved by adequately training the service providers to respond to users’ needs with empathy and assurance [31]. Another issue to consider is the specific nature of the COVID-19 pandemic, where normal health care delivery was disrupted [54], and it was not easy to reach trained medical professionals [55]. During the pandemic, a protocolized management of COVID-19 was approved nationally [56] and was implemented through SB. However, in normal circumstances, people might prefer reaching out to trusted health facilities and doctors, as trust between provider and patient has been shown to be a crucial component of adherence to treatment [57,58]. In Bangladesh, previous studies have shown that preference for trusted health care professionals and lack of trust on telemedicine doctors created a significant barrier in the use of teleconsultation.

Though telemedicine services have improved and technological development has risen in recent years, SB services are only limited to teleconsultation and e-prescription [51]. In addition, prescriptions provided by telemedicine are limited to adhere with health regulations [59]. Despite its limitations, SB has proven to be an important source of health information during emergency situations where normal health care delivery is disrupted [60]. The low-cost access to professional medical advice provided has the potential to bolster health care provision in the future. It is important, therefore, that the SB services are monitored to ensure the quality of service provision and address the gaps. It is also important to think through how best to optimally use SB services to enhance health care access and use during nonpandemic times. In this regard, it is important to address the existing digital divide that is apparent in the use of SB. Most of the callers of SB were better educated, young, male, and urban residents. It will be important to address the barriers faced by population groups that do not use SB services.

Overall, the findings of our study suggest that decision makers should consider “service quality” as an important strategic objective to ensure positive satisfaction and continuance intentions. Good-quality teleconsultation services can help health providers enhance health care coverage during pandemics. Policy makers can develop a tailored regulatory framework to ensure the quality of SB services. In this regard, investments in robust telecommunication infrastructure, especially in rural and hard-to-reach areas, are important to enhance technology accessibility and connectivity. Although teleconsultation services such as SB cannot be an alternative to regular health care, there are important lessons to be learnt from SB. The SB physicians were trained thoroughly in dealing with patients with professionalism and empathy, which led to increased patient satisfaction. As patient satisfaction was an important determinant of intention to use the service in the future, there should be constant efforts made to train health care providers to prioritize patient-centered care, incorporate patient feedback, and ensure user-friendly and accessible telemedicine platforms. Finally, a commitment to research and development, including resource allocation for studies on telemedicine’s impact on user satisfaction, needs to be made to foster innovation and improvement of teleconsultation services.

The strength of the study is the use of a validated service quality model to understand the impact of service quality on patient satisfaction and intention to continue using the service [30]. Another strength of this study is its focus on pandemic health care needs. The study had a few limitations: first, the context of the study is a single provider, and the study applies to the pandemic context. The findings regarding SB’s acceptability may not be generalizable to a nonpandemic context. In the study, we were only able to measure the intention to continue to use the SB services rather than the actual continuance. In the future, longitudinal studies could be conducted to follow up and measure service quality and satisfaction in relationship to the actual continued use of the application.

### Conclusions

This study used a validated framework to evaluate national teleconsultation services in Bangladesh during the COVID-19 pandemic. The findings of this study imply that service quality in health care is an important factor to improve user satisfaction. This study is significant in the context of Bangladesh and other LMICs, where there are human resource constraints in health care, which can be addressed through teleconsultation services [61]. The key to improving service quality and users’ satisfaction is a combination of an enabling environment and infrastructure that includes a robust platform, trained workforce, data privacy and confidentiality, and proper policies and legislations [30,32]. Additionally, our study findings imply that any teleconsultation service must have a guideline for service providers to ensure that good quality service is provided through teleconsultation. While the advances in telemedicine hold immense promise for improving health services, optimal benefits can be availed if service quality is adequately evaluated and monitored.

## Supplementary material

10.2196/46566 Multimedia Appendix 1 Definition of the constructs.

10.2196/46566 Multimedia Appendix 2 Questionnaire for teleconsultation services user.

## Abbreviations

CFA: confirmatory factor analysis
LMIC: low- and middle-income country
mHealth: mobile health
PLS: partial least squares
SEM: structural equation modeling

## References

[1] COVID-19 significantly impacts health services for noncommunicable diseases. World Health Organization.

[2] Craig J, Patterson V (2005). Introduction to the practice of telemedicine. J Telemed Telecare.

[3] Howitt P, Darzi A, Yang GZ (2012). Technologies for global health. Lancet.

[4] Ohannessian R, Duong TA, Odone A (2020). Global telemedicine implementation and integration within health systems to fight the COVID-19 pandemic: a call to action. JMIR Public Health Surveill.

[5] Ramaswamy A, Yu M, Drangsholt S (2020). Patient satisfaction with telemedicine during the COVID-19 pandemic: retrospective cohort study. J Med Internet Res.

[6] Akseer N, Kandru G, Keats EC, Bhutta ZA (2020). COVID-19 pandemic and mitigation strategies: implications for maternal and child health and nutrition. Am J Clin Nutr.

[7] Kaye AD, Okeagu CN, Pham AD (2021). Economic impact of COVID-19 pandemic on healthcare facilities and systems: international perspectives. Best Pract Res Clin Anaesthesiol.

[8] Haldane V, Zhang Z, Abbas RF (2020). National primary care responses to COVID-19: a rapid review of the literature. BMJ Open.

[9] Humayun A, Shahabuddin S, Afzal S, Malik AA, Atique S, Iqbal U (2021). Healthcare strategies and initiatives about COVID19 in Pakistan: telemedicine a way to look forward. Comput Methods Programs Biomed Update.

[10] Peiris D, Sharma M, Praveen D, Bitton A, Bresick G, Coffman M (2021). Strengthening primary health care in the COVID-19 era: a review of best practices to inform health system responses in low- and middle-income countries. WHO South East Asia J Public Health.

[11] Nit B, Kobashi Y, Vory S (2021). The introduction of telemedicine is required immediately in Cambodia: barriers and lessons from COVID-19. J Glob Health.

[12] Chowdhury SR, Sunna TC, Ahmed S (2021). Telemedicine is an important aspect of healthcare services amid COVID‐19 outbreak: its barriers in Bangladesh and strategies to overcome. Int J Health Plann Manage.

[13] Malhotra N, Sakthivel P, Gupta N, Nischal N, Ish P (2022). Telemedicine: a new normal in COVID era; perspective from a developing nation. Postgrad Med J.

[14] (2015). Bangladesh health system review. World Health Organization.

[15] Khan MAH, Cruz V de O, Azad AK (2019). Bangladesh’s digital health journey: reflections on a decade of quiet revolution. WHO South East Asia J Public Health.

[16] Ahmed R (2020). Bangladesh fares poorly in use of technology to fight COVID-19. Prothomalo.

[17] Biswas RK, Huq S, Afiaz A, Khan HTA (2020). A systematic assessment on COVID-19 preparedness and transition strategy in Bangladesh. J Eval Clin Pract.

[18] Bali S (2019). Telehealth.

[19] Hong Z, Li N, Li D (2020). Telemedicine during the COVID-19 pandemic: experiences from Western China. J Med Internet Res.

[20] Hoque R, Sorwar G (2017). Understanding factors influencing the adoption of mHealth by the elderly: an extension of the UTAUT model. Int J Med Inform.

[21] Abdel-Wahab M, Rosenblatt E, Prajogi B, Zubizarretta E, Mikhail M (2020). Opportunities in telemedicine, lessons learned after COVID-19 and the way into the future. Int J Radiat Oncol Biol Phys.

[22] Duggirala M, Rajendran C, Anantharaman RN (2008). Patient‐perceived dimensions of total quality service in healthcare. Benchmarking.

[23] (2020). Implementing telemedicine services during COVID-19: guiding principles and considerations for a stepwise approach. World Health Organization.

[24] Oppong E, Hinson RE, Adeola O, Muritala O, Kosiba JP (2021). The effect of mobile health service quality on user satisfaction and continual usage. Total Qual Manag Bus Excell.

[25] Tweena SN, Oriade A, Mahdi M (2021). An empirical investigation of the impact of service quality on satisfaction, usage behaviors, and quality of life in m-health service context: a case of Bangladesh. Glob Conf on Serv Mgmt.

[26] Lee WI, Figueredo NM (2021). Exploring the perspective of service quality in mHealth services during the COVID-19 pandemic. Int J Econ Manag Eng.

[27] Khatun F, Hanifi SMA, Iqbal M (2014). Prospects of mHealth services in Bangladesh: recent evidence from Chakaria. PLoS One.

[28] Sowon K, Chigona W Trust in mhealth: how do maternal health clients accept and use mhealth interventions?.

[29] Preaux J, Casadesús M, Bernardo M (2023). A conceptual model to evaluate service quality of direct-to-consumer telemedicine consultation from patient perspective. Telemed J E Health.

[30] Akter S, D’Ambra J, Ray P (2010). Service quality of mHealth platforms: development and validation of a hierarchical model using PLS. Electron Markets.

[31] Akter S, D’Ambra J, Ray P (2013). Development and validation of an instrument to measure user perceived service quality of mHealth. Inf Manag.

[32] Akter S, D’Ambra J, Ray P User perceived service quality of mhealth services in developing countries.

[33] Adam Mahmood MO, Burn JM, Gemoets LA, Jacquez C (2000). Variables affecting information technology end-user satisfaction: a meta-analysis of the empirical literature. Int J Hum Comput Stud.

[34] Zviran M, Erlich Z (2003). Measuring IS user satisfaction: review and implications. Commun Assoc Inf Syst.

[35] Cronin JJ Jr, Taylor SA (1992). Measuring service quality: a reexamination and extension. J Mark.

[36] Dabholkar PA, Shepherd CD, Thorpe DI (2000). A comprehensive framework for service quality: an investigation of critical conceptual and measurement issues through a longitudinal study. J Retailing.

[37] A-priori sample size calculator for structural equation models. DanielSoper.

[38] Bentler P (2006). EQS Structural Equations Model Program Manual.

[39] Chin DN (2001). Empirical evaluation of user models and user-adapted systems. User Model User-Adapt Interact.

[40] Chin WW (1998). Modern Methods for Business Research.

[41] Tenenhaus M, Vinzi VE, Chatelin YM, Lauro C (2005). PLS path modeling. Comput Stat Data Anal.

[42] Wetzels M, Odekerken G, van Oppen C (2009). Using PLS path modeling for assessing hierarchical construct models: guidelines and empirical illustration. MIS Q.

[43] Fornell C, Larcker DF (1981). Structural equation models with unobservable variables and measurement error: algebra and statistics. The University of Michigan Library.

[44] Alam MZ, Khanam L (2023). Understanding the determinants of adoption of mHealth services among older women’s perspective in Bangladesh. Int J Pharm Healthc Mark.

[45] Khatun F, Heywood AE, Ray PK, Hanifi SMA, Bhuiya A, Liaw ST (2015). Determinants of readiness to adopt mHealth in a rural community of Bangladesh. Int J Med Inform.

[46] Amin R, Hossain MA, Uddin MM, Jony MTI, Kim M (2022). Stimuli influencing engagement, satisfaction, and intention to use telemedicine services: an integrative model. Healthcare (Basel).

[47] Khatun F, Heywood AE, Ray PK, Bhuiya A, Liaw ST (2016). Community readiness for adopting mHealth in rural Bangladesh: a qualitative exploration. Int J Med Inform.

[48] Uzir MUH, Al Halbusi H, Lim R (2021). Applied artificial intelligence and user satisfaction: smartwatch usage for healthcare in Bangladesh during COVID-19. Technol Soc.

[49] Zobair KM, Sanzogni L, Houghton L, Islam MZ (2021). Forecasting care seekers satisfaction with telemedicine using machine learning and structural equation modeling. PLoS ONE.

[50] Birkmeyer S, Wirtz BW, Langer PF (2021). Determinants of mHealth success: an empirical investigation of the user perspective. Int J Inf Manage.

[51] Khatun F, Ahmed NU, Rahman H (2023). The promise of teleconsultation in the era of pandemic: a case from Bangladesh. Telemed J E Health.

[52] Wang J, Cao Y (2022). Factors influencing continuous intention to use telemedicine after the COVID-19 pandemic in China: an extended technology acceptance model. Open J Soc Sci.

[53] Amin R, Hossain MA, Uddin MM, Jony MTI, Kim M (2022). Stimuli influencing engagement, satisfaction, and intention to use telemedicine services: an integrative model. Healthcare (Basel).

[54] Rahman M, Rahman A, Siddique KB, Islam R, Ur Rahim MM (2023). Impact of COVID-19 on essential health care of rural people in Northern Bangladesh: a cross-sectional study. Int J Transl Med Res Public Health.

[55] Razu SR, Yasmin T, Arif TB (2021). Challenges faced by healthcare professionals during the COVID-19 pandemic: a qualitative inquiry from Bangladesh. Front Public Health.

[56] Guidelines and rules [Article in Bangla]. Directorate General of Health Services (DGHS).

[57] Mohiuddin AK (2020). An extensive review of patient satisfaction with healthcare services in Bangladesh. Patient Exp J.

[58] Haque MM (2021). Policy Response, Local Service Delivery, and Governance in Bangladesh.

[59] (2020). Telemedicine guidelines. Bangladesh Medical & Dental Council.

[60] Uddin Ahmed N, Rahman M, Shekhor Roy S (2022). Impact of telehealth services through “Shastho Batayon 16263” for tackling the COVID-19 pandemic in Bangladesh. East J Healthc.

[61] Motamarri S, Akter S, Ray P, Tseng CL mHealth: a better alternative for healthcare in developing countries.

