# Supplementary material for: Users’ Perceived Service Quality of National Telemedicine Services During the COVID-19 Pandemic in Bangladesh: Cross-Sectional Study
Source: JMIR Hum Factors. 2024 Dec 23;11:e46566. doi: 10.2196/46566 (PMC12264782; doi:10.2196/46566)
Supplement: Multimedia Appendix 1 [file humanfactors-v11-e46566-s001.docx]

PQ (Platform quality): This refers to the perceptions of the service recipients about the technical quality of the telehealth service platform. [40, 50].

IQ (Interaction quality): The degree to which a user perceives the quality of the provided telehealth service in terms of service provider’s knowledge, competence as well as their promptness in delivering the service and attentiveness to client’s needs [40, 51, 52].

OQ (Outcome quality): This indicates the cumulative benefits that the clients get after using the telehealth service [40, 50].

Service quality (SQ): This refers to the client’s impression on performance of the received service [53].

User satisfaction (SAT): This reflects user’s feelings and attitude towards the various components of the service received [54].

Intention to continue (IQ): This reflects the user’s behavioural pattern indicating their willingness to continue using the service [55].
