# Supplementary material for: Users’ Perceived Service Quality of National Telemedicine Services During the COVID-19 Pandemic in Bangladesh: Cross-Sectional Study
Source: JMIR Hum Factors. 2024 Dec 23;11:e46566. doi: 10.2196/46566 (PMC12264782; doi:10.2196/46566)
Supplement: Multimedia Appendix 2 [file humanfactors-v11-e46566-s002.docx]

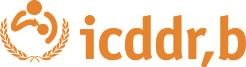


Questionnaire for Tele-consultation services user (English)

Protocol Title: Tracking clinically suspect COVID-19 cases using existing national teleconsultation services in Bangladesh

Interviewer name ______________________

| 101 | Age | (in complete year) | [__\|__] |
| --- | --- | --- | --- |
| 102 | Sex | Male= 1  Female= 2 | [__] |
| 103 | For whom did you call *Shastho Batayon* (SB)? | Myself= 1  Others= 2 | [__]  If 1, skip to  106 |
| If called for others, please provide detailed information about that person | | |  |
| 104 | Age | (in complete year/ months in case of infant) | [__\|__] |

Study ID (SID): |__|__|__|__|

Date: _ _/_ _/_ _ (DD/MM/YY)

Interview Starting Time: [__|__] : [__|__] AM/PM

# Interview End Time: [__|__] : [__|__] AM/PM

Result of the Interview: Complete =1, Incomplete=2, Absent=3

1. Basic Demographics

| 105 | Sex | Male= 1 Female= 2 | [__] |
| --- | --- | --- | --- |
|  | All the following information’s are of the person for whom care was sought | | |
| 106 | Area of residence | Rural= 1  Semi urban= 2  Urban= 3 | [__] |
| 107 | Education | (completed years of education) | [__\|__] |
| 108 | Ethnicity | Bengali= 1  Other= 2 | [__] if others specify here: ---------  - |
| 109 | Marital status | Unmarried= 1  Married= 2  Widow= 3  Divorced= 4 | [__] |
| 110 | Occupation (Self/Guardian) | Business or self-employed= 1  Unskilled manual labour= 2  Skilled manual labour= 3  Professional= 4  Farmer= 5  Student= 6  At home doing housework= 7  Unemployed= 8  Other (specify)= 9 | [__]  If others specify here:---------  - |

| 111 | Number of current household members |  | [__\|__] |
| --- | --- | --- | --- |
| 112 | Number of rooms in household |  | [__\|__] |
| 113 | Household’s average monthly expenses in a typical month (taka/month) | Write in complete figure | [__\|__\|__] |

1. Items development for service quality constructs please chose any of the mentioned option as your answer

Section A: Shastho Batayon Service quality

|  | Dimension | Statement | Strongly agree | Agree | Neutral | Disagree | Strongly disagree |  |
| --- | --- | --- | --- | --- | --- | --- | --- | --- |
| A11 | Systems  Reliability | This service platform works smoothly | 5 | 4 | 3 | 2 | 1 | [__] |
| A12 |  | This service platform performs reliably | 5 | 4 | 3 | 2 | 1 | [__] |
| A13 |  | This service platform is dependable | 5 | 4 | 3 | 2 | 1 | [__] |
| A21 | Systems  Availability | This platform is always available | 5 | 4 | 3 | 2 | 1 | [__] |
| A22 |  | I can receive medical service right away | 5 | 4 | 3 | 2 | 1 | [__] |
| A23 |  | It does not have long waiting time | 5 | 4 | 3 | 2 | 1 | [__] |
| A31 | Systems Efficiency | This service platform can be adapted to meet variety of needs | 5 | 4 | 3 | 2 | 1 | [__] |
| A32 |  | This service platform can flexibly adjust to new conditions | 5 | 4 | 3 | 2 | 1 | [__] |

| A33 |  | This service platform is well organized | 5 | 4 | 3 | 2 | 1 | [__] |
| --- | --- | --- | --- | --- | --- | --- | --- | --- |
| A41 | Systems Privacy | This platform protects information about my personal problems | 5 | 4 | 3 | 2 | 1 | [__] |
| A42 |  | This platform does not share my personal information with others | 5 | 4 | 3 | 2 | 1 | [__] |
| A43 |  | This platform offers me a meaningful guarantee | 5 | 4 | 3 | 2 | 1 | [__] |
| A51 | Responsivene  ss | Physicians of SB platform provide prompt service | 5 | 4 | 3 | 2 | 1 | [__] |
| A52 |  | Physicians are never too busy to respond to my requests | 5 | 4 | 3 | 2 | 1 | [__] |
| A53 |  | Physicians are willing to help me | 5 | 4 | 3 | 2 | 1 | [__] |
| A54 |  | They provide the service by a certain time | 5 | 4 | 3 | 2 | 1 | [__] |
| A61 | Assurance | The behaviour of physicians instils confidence in me | 5 | 4 | 3 | 2 | 1 | [__] |
| A62 |  | I feel safe while consulting with physicians | 5 | 4 | 3 | 2 | 1 | [__] |
| A63 |  | Physicians have the knowledge to answer my questions | 5 | 4 | 3 | 2 | 1 | [__] |
| A64 |  | Physicians are competent in providing service | 5 | 4 | 3 | 2 | 1 | [__] |
| A71 | Empathy | Physicians give me personal attention | 5 | 4 | 3 | 2 | 1 | [__] |
| A72 |  | Physicians give me individual care | 5 | 4 | 3 | 2 | 1 | [__] |
| A73 |  | Physicians understand my specific needs | 5 | 4 | 3 | 2 | 1 | [__] |
| A74 |  | Physicians have my best interests at heart | 5 | 4 | 3 | 2 | 1 | [__] |
| A81 | Functional Benefit | It serves my purpose very well | 5 | 4 | 3 | 2 | 1 | [__] |
| A82 |  | I believe having service from this platform has been worthwhile | 5 | 4 | 3 | 2 | 1 | [__] |
| A83 |  | It is convenient to use this health service | 5 | 4 | 3 | 2 | 1 | [__] |
| A84 |  | Overall this service is useful to me | 5 | 4 | 3 | 2 | 1 | [__] |
| A91 | Emotional  Benefit | I feel positive using this health service | 5 | 4 | 3 | 2 | 1 | [__] |
| A92 |  | I feel hopeful as a result of having this service | 5 | 4 | 3 | 2 | 1 | [__] |
| A93 |  | I feel encouraged as a result of having this service | 5 | 4 | 3 | 2 | 1 | [__] |

Section B: Intention to continue

|  | Statement | Strongly agree | Agree | Neutral | Disagree | Strongly disagree |  |
| --- | --- | --- | --- | --- | --- | --- | --- |
| B01 | I intend to continue using mobile health service to get medical information services | 5 | 4 | 3 | 2 | 1 | [__] |
| B02 | My intention is to continue using this service rather than use any alternative means (e.g. local clinics) | 5 | 4 | 3 | 2 | 1 | [__] |
| B03 | I will not discontinue my use of this service. | 5 | 4 | 3 | 2 | 1 | [__] |

Section C: Satisfaction

| C01 | Generally, how do you feel for this telemedicine (SB) service for your primary health care | Delighted | Contented | Satisfied | Dissatisfied | Frustrated | Absolutely terrible |  |
| --- | --- | --- | --- | --- | --- | --- | --- | --- |
|  |  | 6 | 5 | 4 | 3 | 2 | 1 | [__] |
| C02 | Have you shared your experiences with SB with family and friends? | Yes = 1  No = 2 |  |  |  |  |  | [__] |

*Thank the participant for his/her cooperation*
